# RS3PE as a Paraneoplastic Manifestation of Acute Myeloid Leukaemia With IDH1 and KMT2A Mutations: A Case Report

**DOI:** 10.7759/cureus.113788

**Published:** 2026-08-01

**Authors:** Ibrahim Saleh, Wesam Gouda

**Affiliations:** 1 Rheumatology, Ministry of Health of Kuwait, Kuwait City, KWT; 2 Rheumatology, Al-Azhar University, Cairo, EGY

**Keywords:** acute myeloid leukemia, paraneoplastic syndrome, pitting edema, rs3pe, seronegative arthritis, synovitis

## Abstract

Remitting seronegative symmetrical synovitis with pitting oedema (RS3PE) is an inflammatory syndrome of older adults that has been increasingly associated with underlying malignancy.

A 67-year-old woman presented with abrupt-onset symmetrical polyarthritis and marked bilateral pitting oedema. Laboratory investigations showed elevated inflammatory markers and normocytic anaemia, with negative rheumatoid serology. She achieved complete remission with low-dose corticosteroids. Months after steroid withdrawal, inflammatory arthritis recurred alongside worsening anaemia (haemoglobin 7.6 g/dL), leukopenia (white blood cell count 2.5 × 10⁹/L; absolute neutrophil count 0.38 × 10⁹/L), platelet count of 150 × 10⁹/L, and circulating blast cells. Bone marrow biopsy confirmed acute myeloid leukaemia (AML) with 33%-40% blasts.

Molecular analysis identified *IDH1* and *KMT2A* mutations. Treatment with azacitidine-venetoclax resulted in complete remission of both haematological disease and rheumatologic manifestations without corticosteroids.

RS3PE may represent an early paraneoplastic manifestation of AML. Recurrence of symptoms after steroid withdrawal should prompt evaluation for malignancy. Molecular characterisation may provide additional insight into disease mechanisms and treatment response.

## Introduction

Remitting seronegative symmetrical synovitis with pitting oedema (RS3PE) was first described in 1985 as a distinct inflammatory syndrome affecting older individuals [1]. It is characterised by abrupt-onset symmetrical synovitis, distal pitting oedema, seronegativity, and dramatic responsiveness to low-dose corticosteroids [2,3].

Diagnostic criteria typically include age greater than 50 years, acute symmetrical synovitis, pitting oedema of the hands and/or feet, negative rheumatoid factor, and absence of erosive changes on imaging [2,3].

Although initially considered benign, subsequent studies have demonstrated malignancy associations in approximately 10%-30% of cases [4]. Haematologic malignancies, including myelodysplastic syndromes and acute leukaemias, represent a significant proportion of these associations [5,6]. Elevated vascular endothelial growth factor (VEGF) has been implicated in the pathogenesis of RS3PE and may explain the characteristic oedema through increased vascular permeability.

Reports linking RS3PE to AML remain limited, particularly those including molecular characterisation [6]. We present a molecularly defined AML-associated RS3PE case with complete remission following leukaemia-directed therapy.

Paraneoplastic rheumatic syndromes are immune-mediated manifestations triggered by underlying malignancy and may precede tumour diagnosis. Recognition of these syndromes is clinically critical, as treatment of the primary neoplasm often results in resolution of inflammatory symptoms.

## Case presentation

A 67-year-old woman with a medical history significant for hypertension, type 2 diabetes mellitus, and hypothyroidism presented with a two-month history of abrupt-onset symmetrical inflammatory polyarthritis. She reported pain, prolonged morning stiffness exceeding one hour, and progressive swelling involving the wrists, metacarpophalangeal and proximal interphalangeal joints, knees, and ankles.

**Figure 1:**
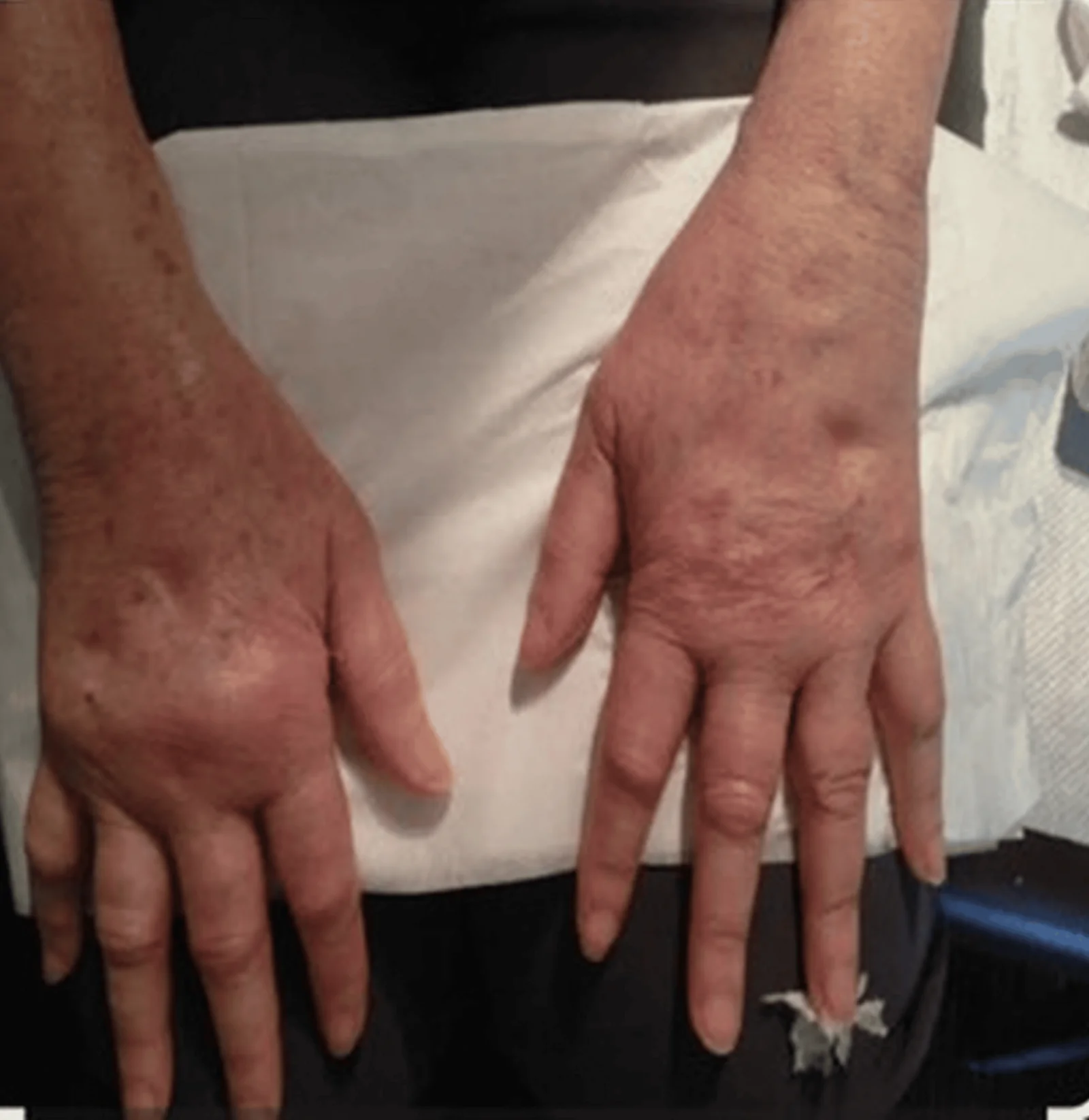
Bilateral hand pitting oedema and synovitis before treatment

**Figure 2:**
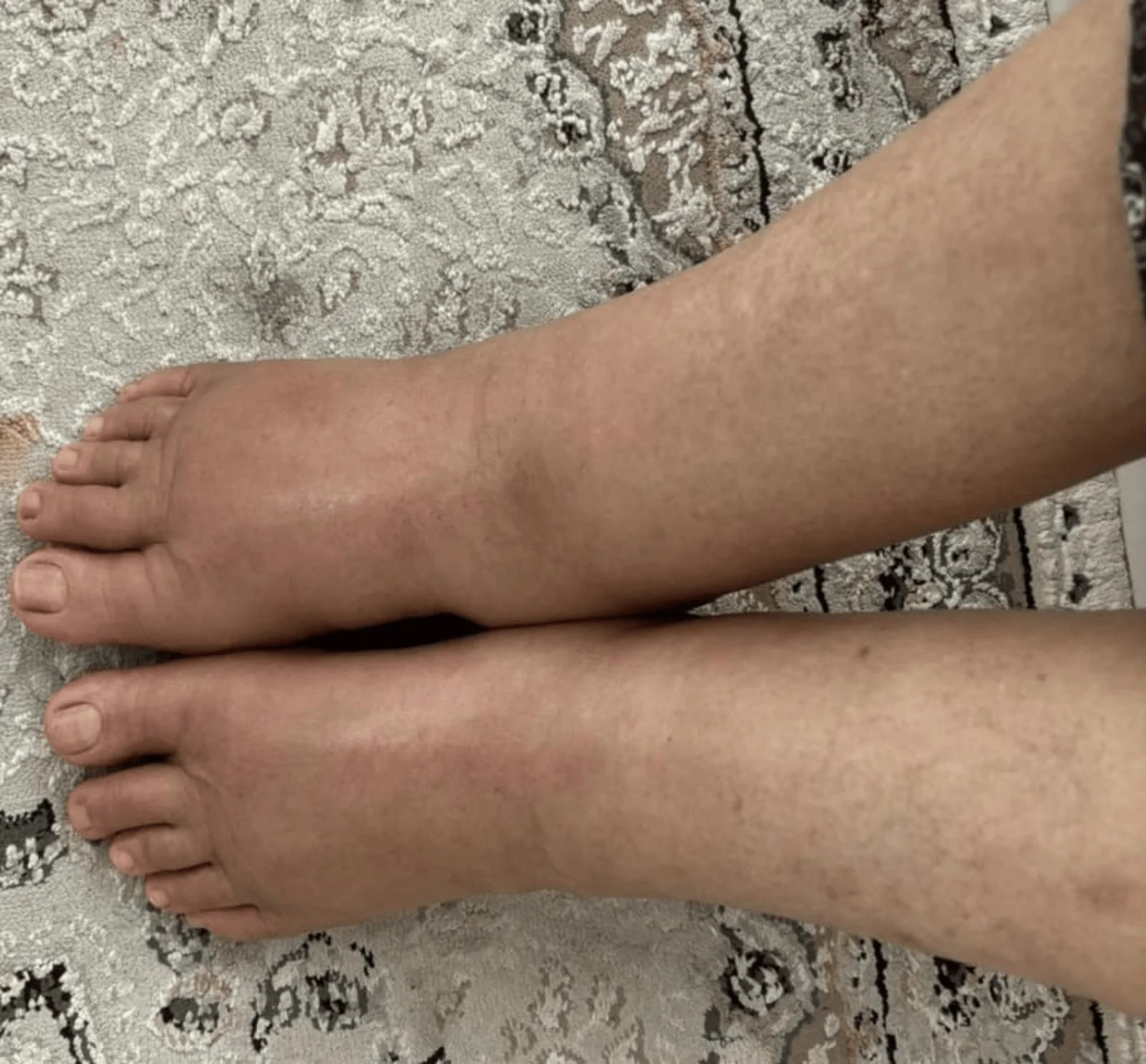
Foot oedema before treatment

On examination, marked bilateral pitting oedema was noted over the dorsum of the hands and extending to the lower extremities. Active synovitis was evident in multiple small and large joints, with reduced grip strength secondary to swelling. No lymphadenopathy or hepatosplenomegaly was detected. As shown in Figures 1-2, marked bilateral pitting oedema of the hands and feet was observed.

Laboratory investigations demonstrated an ESR of 85 mm/hr and a CRP level of 59 mg/L. Complete blood count at presentation revealed a white blood cell count of 8 × 10⁹/L, haemoglobin of 10 g/dL, and a platelet count of 250 × 10⁹/L. Rheumatoid factor, anti-cyclic citrullinated peptide antibodies, and antinuclear antibodies were negative.

The diagnosis fulfilled the original McCarty criteria for RS3PE, including age above 50 years, acute symmetrical synovitis, characteristic pitting oedema, seronegativity, and marked corticosteroid responsiveness [1,3].

Key distinguishing features are summarised in Table 1.

**Table 1:** Differential diagnosis RF/ACPA: rheumatoid factor/anticitrullinated protein antibodies; RS3PE: remitting seronegative symmetrical synovitis with pitting oedema

Feature	RS3PE	Rheumatoid Arthritis	Polymyalgia Rheumatica
Age >50	Yes	Variable	Yes
Acute onset	Yes	Gradual	Subacute
RF/ACPA	Negative	Often positive	Negative
Pitting oedema	Characteristic	Rare	Rare
Erosions	Absent	Present	Absent
Steroid response	Dramatic	Variable	Good

However, the presence of characteristic pitting oedema, seronegativity, and dramatic corticosteroid responsiveness supported RS3PE over alternative diagnoses [2]. Prednisolone 15 mg daily was initiated, resulting in rapid and complete remission within four weeks.

Corticosteroids were tapered and discontinued after sustained improvement. Several months later, inflammatory arthritis recurred, accompanied by fatigue. Recurrence of inflammatory manifestations in parallel with evolving haematologic abnormalities raised suspicion for an underlying clonal process.

Repeat laboratory testing demonstrated worsening anaemia (haemoglobin 7.6 g/dL), leukopenia (white blood cell count 2.5 × 10⁹/L; absolute neutrophil count 0.38 × 10⁹/L (15%)), platelet count of 150 × 10⁹/L, and circulating blast cells. Bone marrow biopsy revealed 33%-40% myeloblasts consistent with acute myeloid leukaemia (AML) according to WHO classification [7]. Cytogenetic analysis showed a normal karyotype, and next-generation sequencing identified *IDH1* and *KMT2A* mutations.

The patient was treated with azacitidine 75 mg/m²/day administered intravenously for seven consecutive days per 28-day cycle, combined with oral venetoclax (400 mg daily following dose ramp-up) for 14 days per cycle [8].

The patient completed six cycles of combination therapy and achieved haematologic remission. At six months of follow-up, complete resolution of synovitis and pitting oedema was maintained without reintroduction of corticosteroids. Mild treatment-related fatigue was reported, but no major infectious or haematologic complications occurred. No recurrence of inflammatory symptoms was observed.

**Figure 3:**
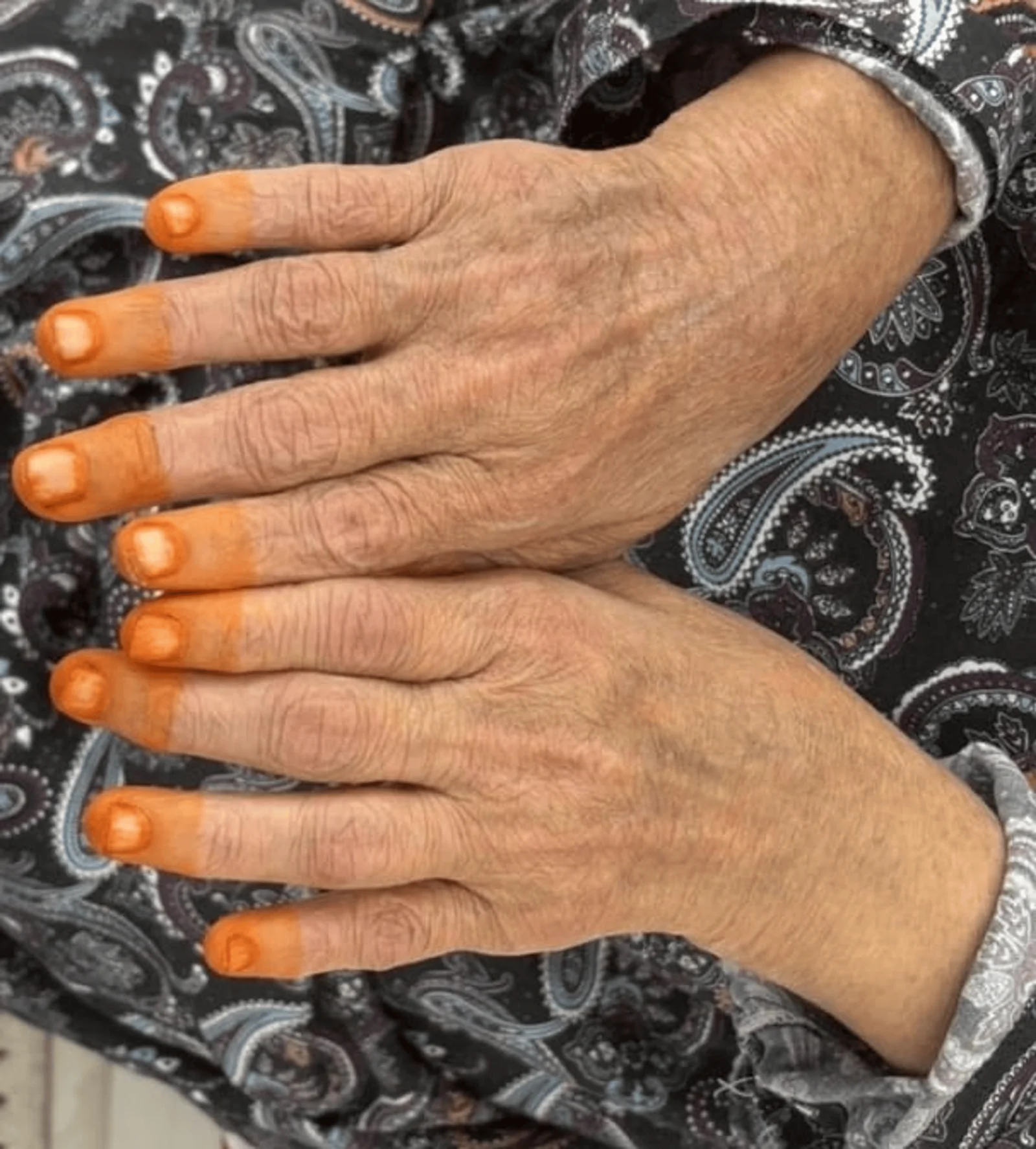
Resolution of bilateral hand pitting oedema and synovitis after treatment

**Figure 4:**
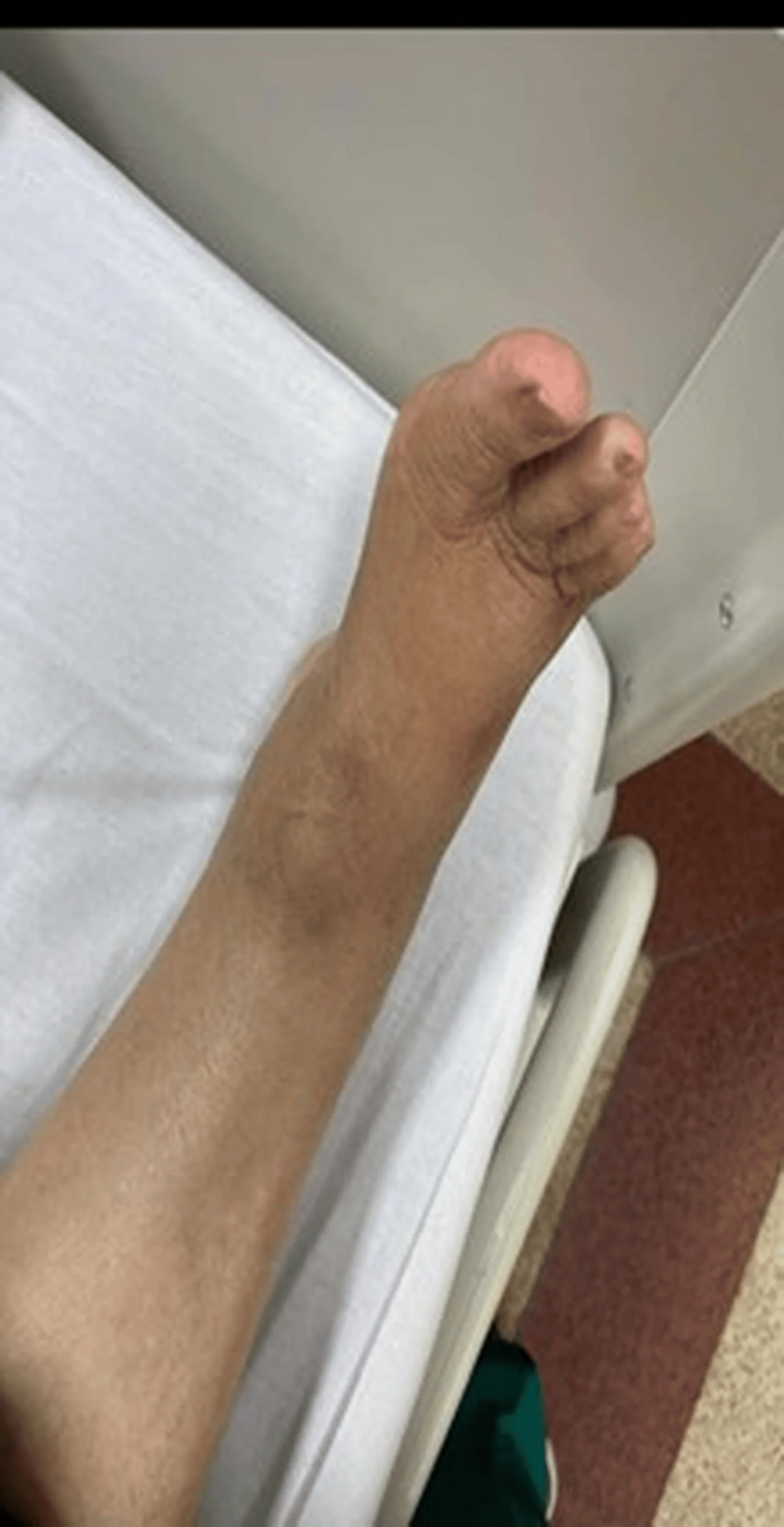
Resolution of lower limb oedema after treatment, consistent with clinical remission of RS3PE RS3PE: remitting seronegative symmetrical synovitis with pitting oedema

Follow-up clinical and photographic documentation demonstrated complete resolution of synovitis and pitting oedema after azacitidine-venetoclax therapy (Figures 3-4).

## Discussion

RS3PE is increasingly recognised as a paraneoplastic syndrome, particularly in older patients. Although it typically demonstrates dramatic responsiveness to corticosteroids, this response does not exclude an underlying malignancy. In some cases, initial improvement may delay the diagnosis of an evolving neoplastic process.

This case fulfils key criteria for a paraneoplastic rheumatic syndrome, including elderly onset, temporal association with malignancy, and complete resolution of symptoms following cancer-directed therapy without corticosteroids. The recurrence of arthritis alongside emerging haematologic abnormalities supports a possible paraneoplastic association rather than simple coincidental coexistence.

Previously reported cases are summarised in Table 2.

**Table 2:** AML-associated RS3PE cases AML: acute myeloid leukaemia; RS3PE: remitting seronegative symmetrical synovitis with pitting oedema

Author	Year	RS3PE Preceded AML	Resolution After Chemotherapy
Chiappetta and Gruber [6]	2005	Yes	Yes
Present Case	2026	Yes	Complete

Haematologic malignancies represent a significant subset of these associations, including myelodysplastic syndromes, chronic leukaemias, and AML [5,6]. In the limited published literature, RS3PE preceded the diagnosis of AML by months to years, suggesting that inflammatory manifestations may represent an early paraneoplastic signal [6].

In the present case, arthritis relapse following corticosteroid withdrawal coincided with haematologic deterioration and blast appearance in peripheral blood. This temporal sequence argues against isolated autoimmune relapse and instead suggests evolving clonal haematologic disease.

The complete disappearance of inflammatory manifestations after azacitidine-venetoclax therapy provides further support for a possible paraneoplastic association. Similar patterns have been observed in other malignancy-associated rheumatic syndromes, in which adequate tumour control leads to resolution of systemic inflammatory features [9]. Post-treatment complete blood counts demonstrated normalisation of haematological parameters, with a white blood cell count of 8 × 10⁹/L, haemoglobin of 12 g/dL, and platelet count of 150 × 10⁹/L. A follow-up bone marrow examination was not performed because the patient was clinically improved, of advanced age, and no clinical indication for repeat bone marrow assessment was present.

**Figure 5:**
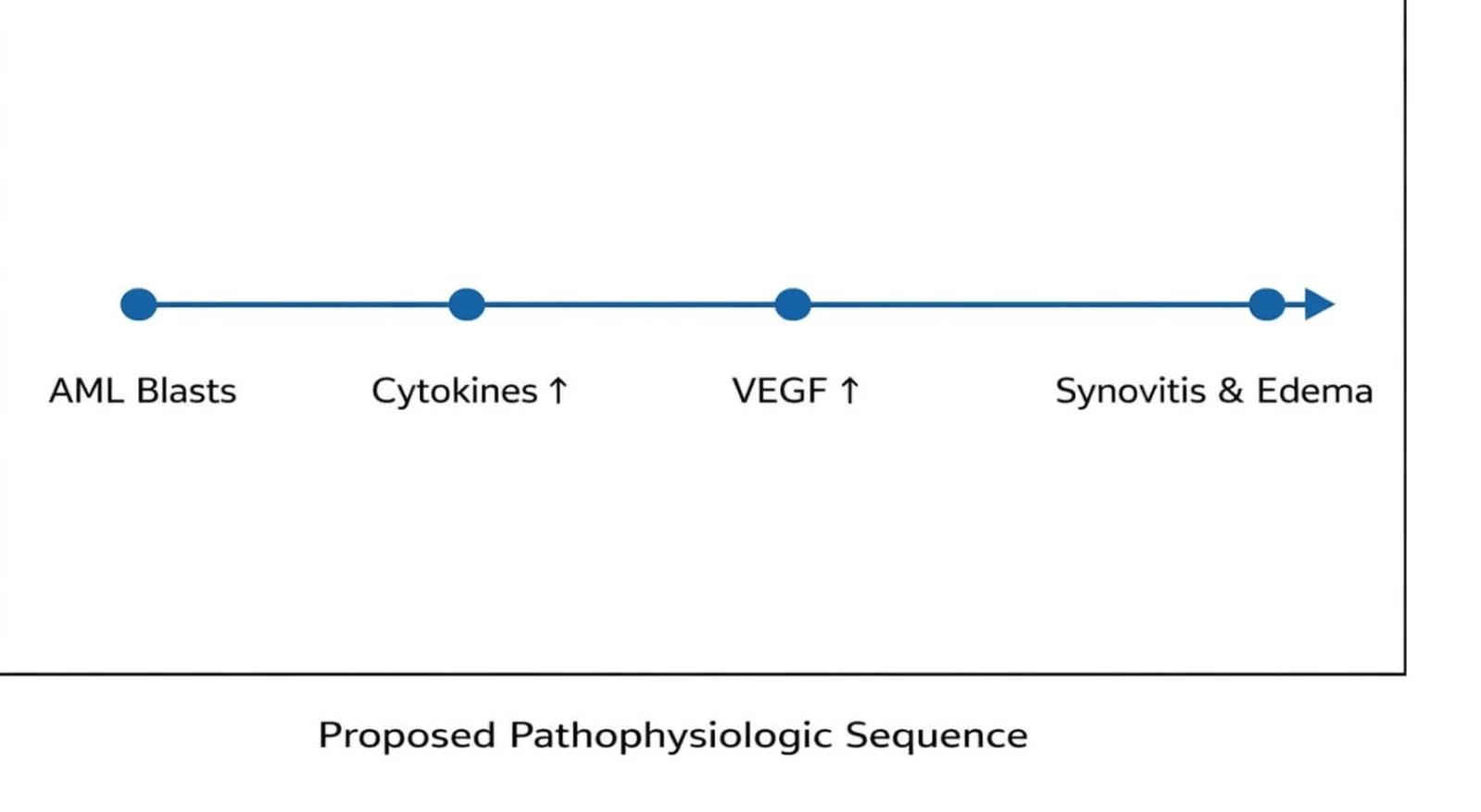
Hypothetical paraneoplastic mechanism linking AML, cytokine upregulation, VEGF signalling, and RS3PE This schematic was created by the authors based on the current literature. AML: acute myeloid leukaemia; RS3PE: remitting seronegative symmetrical synovitis with pitting oedema; VEGF: vascular endothelial growth factor

VEGF-mediated vascular permeability is believed to play a central role in RS3PE pathogenesis (Figure 5).

Elevated VEGF levels have been documented in affected patients and correlate with the severity of oedema. Leukaemic blasts can produce pro-inflammatory cytokines, including IL-6 and VEGF, potentially creating a systemic inflammatory milieu conducive to synovial inflammation and capillary leak. However, serum VEGF and inflammatory cytokine levels were not measured in the present patient. Therefore, the proposed pathogenic mechanism remains hypothetical and should be interpreted cautiously.

The identification of *IDH1* and *KMT2A* mutations adds molecular depth to this case. *IDH1* mutations are associated with altered cellular metabolism and epigenetic dysregulation in AML. *KMT2A* abnormalities are linked to aggressive leukaemic behaviour and transcriptional activation pathways. Because detailed molecular sequencing data (variant allele frequency, transcript/protein change, and sequencing platform) were unavailable from the referral laboratory, further molecular characterisation could not be provided. Although their direct contribution to paraneoplastic inflammatory syndromes remains speculative, molecular heterogeneity may influence cytokine expression patterns and systemic immune activation. Importantly, dramatic corticosteroid responsiveness does not exclude malignancy. Several studies have demonstrated that paraneoplastic RS3PE may initially respond to steroids before relapse reveals the underlying neoplasm [4,6]. Therefore, clinical remission should not preclude continued surveillance.

This case has several limitations. Detailed molecular sequencing data, including the exact *IDH1* and *KMT2A* variants, variant allele frequencies, transcript/protein changes, and sequencing platform, were unavailable from the referral laboratory. In addition, serum VEGF and inflammatory cytokine levels were not measured. Although post-treatment complete blood counts demonstrated normalisation of haematological parameters, follow-up bone marrow examination, formal remission assessment, and measurable residual disease evaluation were not performed because the patient showed sustained clinical and haematological improvement and there was no clinical indication for repeat bone marrow assessment.

From a clinical perspective, elderly patients presenting with RS3PE should undergo careful baseline evaluation, including a complete blood count and age-appropriate malignancy screening. Based on the limited published literature and the clinical course of the present case, careful haematological follow-up should be considered in patients with recurrent or atypical RS3PE, particularly in the presence of unexplained anaemia, constitutional symptoms, or other laboratory abnormalities.

In the limited published literature, RS3PE preceded the diagnosis of AML by several months, suggesting that inflammatory manifestations may serve as an early paraneoplastic signal. Resolution of synovitis following leukaemia-directed therapy has been described in isolated case reports, further supporting a possible paraneoplastic association. However, molecular characterisation has rarely been reported, making the present case distinctive in demonstrating *IDH1 *and *KMT2A* mutations in this context [6].

The availability of before-and-after photographic documentation further supports a possible paraneoplastic association, demonstrating complete clinical resolution following leukaemia-directed therapy.

This case contributes to the literature by providing molecular characterisation of AML-associated RS3PE and demonstrating complete remission following hypomethylating agent plus BCL-2 inhibition therapy, further supporting a possible paraneoplastic association.

The clinical course of the patient is summarised in Table 3.

**Table 3:** Clinical timeline RS3PE: remitting seronegative symmetrical synovitis with pitting oedema; AML: acute myeloid leukaemia

Time	Event
Month 0	RS3PE diagnosis
Month 1	Steroid remission
Months 6-10	Relapse
Month 12	AML diagnosis (33%-40% blasts)
Post-chemotherapy	Complete arthritis resolution

## Conclusions

RS3PE may represent an early manifestation of underlying haematologic malignancy. Recurrence of symptoms after corticosteroid withdrawal should prompt evaluation for malignancy, particularly when accompanied by haematologic abnormalities. Molecular characterisation may provide additional insight into pathogenesis and treatment response in malignancy-associated RS3PE.
